# Supplementary material for: WordCommentsAnalyzer: A windows software tool for qualitative research
Source: F1000Res. 2018 Sep 4;7:536. Originally published 2018 May 3. [Version 2] doi: 10.12688/f1000research.14819.2 (PMC6137411; doi:10.12688/f1000research.14819.2)
Supplement: Supplementary file 3 [file f1000research-7-17614-s0002.tgz › b2a79496-4ada-47c4-9682-a99ce867836d.zip!/Eight Iranian Health Journals/Yafteh _53.docx]

© Yafteh  . This work is licensed under CC BY 4.0 (https://creativecommons.org/licenses/by/4.0/).

Volume & Issue: دوره 7 - شماره 4

Title: فراوانی سرطانهای مختلف گوارشی بر اساس آندوسکوپی فوقانی و تحتانی

http://yafte.lums.ac.ir/article-1-1095-fa.html

نتیجه گیری: سرطان مری در بیماران لرستانی مطالعه شده از شایعترین سرطانهای گوارشی می باشد و پس از آن سرطان معده و کولون قرار دارند. مواد و روش ها: مطالعه به صورت توصیفی ـ مقطعی انجام شد و کلیه بیمارانی که از خرم آباد و اطراف در این مرکز تحت آندوسکوپی و کولونوسکوپی قرار گرفته و تحت بیوپسی قرار گرفته بودند بر اساس پاتولوژی نتایج مورد تجزیه و تحلیل قرار گرفته و توزیع سرطانها بر اساس سن و جنس و محل درگیری و نوع پاتولوژی تعیین گردید. یافته ها: از 3088 بیمار آندوسکوپی 408 نفر (2/13%) به سرطانهای مختلف گوارشی مری و معده و کولون مبتلا بودند. شایعترین محل سرطان در بین بیماران را سرطان مری با تعداد 216 نفر (93/52%) تشکیل می داد و سپس به ترتیب سرطان معده و کولون قرار داشتند کلیه سرطانها در مردان شایعتر از زنان بود. شایعترین فرم پاتولوژی در سرطان مری اسکواموس سل کارسینوما (SCC) و در معده و کولون آدنوکارسینوما بود. مقدمه: سرطانهای گوارشی یکی از علل مهم مرگ و میر بیماران در جهان هستند و در کشور ما نیز یکی از علل مهم موربیدتی و مورتالیتی بیماران بوده و از شیوع نسبتا زیادی برخوردارند. شمال کشور ما در کمربند آسیایی سرطان مری قرار داشته و در مناطق دیگر کشور سرطان معده از شایعترین سرطانهای گوارشی می باشد. با توجه به شیوع نسبتا بالای سرطانهای گوارشی در لرستان و مشاهده موارد زیادی سرطان مری این تحقیق با هدف بررسی فراوانی سرطانهای مختلف گوارشی در یکی از مراکز آموزشی درمانی خرم آباد انجام گرفت.

Title: بررسی علل زردی در نوزادان بستری در بیمارستان شهید مدنی خرم اباد در سال 1380

http://yafte.lums.ac.ir/article-1-1096-fa.html

مقدمه: هیپر بیلیروبینمی مشکل شایع نوزادان است که حدود 60% نوزادان ترم و 80% نوزادان پره ترم در هفته اول تولد به آن مبتلا می گردند. کرن ایکتروس یکی از عوارض نادر اما جدی هیپر بیلیروبینمی تشخیص داده نشده می باشد که طی آن بیلیروبین غیر مستقیم در سلول مغزی رسوب کرده و باعث اختلال کار مغز به طور موقتی یا صدمه دائمی مغزی میشود.هدف از این مطالعه بررسی علل زردی نوزادان بستری در بیمارستان شهید مدنی بود نتیجه گیری: در این مطالعه بیشترین علت زردی نوزادان را زردی شیر مادر از نوع زودرس تشکیل می دهد. بنابراین باید به به مادران در خصوص افزایش تعداد دفعات شیردهی و تاثیر آن بر کاهش بروز زردی در نوزادان آموزشهای لازم داده شود. یافته ها: از 123 نوزاد تحت مطاله 72% مذکر و 28% مؤنث بودند . از نظر علل زردی 9/39 % از نوزادان مبتلا به زردی شیر مادر از نوع زودرس بودند که به علت مصرف کم شیر مادر و کاهش دریافت کالری بود.1 /21%دارای ناسازگاری ABO , 4/2% ناسازگاری Rh و 9% نقص G6PD داشتند. 3 /16%سپسیس , 6 /1% مبتلا به عفونت دستگاه ادراری و 7/5 % دچار اسفکسی بودند و 6 /1% انسداد دستگاه گوارش داشتند. 6/1%دچار سفال هماتوم و 8/0%دچار دیابت مادر بودند. از 123 نوزاد, 4/76% با فتوتراپی و 4/23 % با تعویض خون درمان شدند. 4/2 % از نوزادان تحت بررسی قبل از مراجعه به بیمارستان دچار عارضه کرن ایکتروس شده بودند.مواد و روش ها: این مطالعه بصورت توصیفی –مقطعی وآینده نگر انجام شد. نمونه گیری به روش آسان بود. جامعه آماری را کلیه نوزادانی که با تشخیص زردی غیر فیزیولوژیک در سال 1382 در بیمارستان شهید مدنی خرم آباد بستری شدند تشکیل داد.حجم نمونه 123 نوزاد بود متغیرهای مورد بررسی شامل,جنس, سن, سابقه خانوادگی مثبت, تغذیه با شیر مادر, دیابت مادر, زمان شروع زردی بود. با توجه به فول ترم یا نارس بودن نوزاد, سن بعد از تولد و سطح بیلیروبین خون, درمان با فتوتراپی یا تعویض خون انجام شد. اطلاعات لازم بوسیله پرسشنامه جمع آوری و با استفاده از آمار توصیفی تجزیه و تحلیل آماری انجام شد.

Title: رابطه بین سلامت روانی و برخی متغیرهای زمینه ای در دانشجویان دانشگاه لرستان

http://yafte.lums.ac.ir/article-1-1097-fa.html

مقدمه: سلامت روانی یکی از مهمترین عوامل مؤثر در رشد و پرورش استعدادهای دانشجویان محسوب می شود. مطالعات متعدد حاکی از آن است که دانشجویان نیز مبتلا به انواع اختلالات و مشکلات عاطفی هستند. هدف از این مطالعه، مقایسه وضعیت سلامت روانی دانشجویان دانشگاه لرستان در رابطه با برخی متغیرهای فردی و تحصیلی است. مواد و روش ها: در این تحقیق که یک پژوهش توصیفی بوده است، نمونه آماری تحقیق عبارت بود از تعداد 436 نفر دانشجو که با استفاده از روش نمونه گیری طبقه ای به صورت تصادفی انتخاب شده بودند. برای گردآوری داده های مورد نیاز یک پرسشنامه حاوی سوالات مربوط به ویژگیهای جمعیت شناختی و مقیاس سلامت روانی (Mental Health Scale) مورد استفاده قرار گرفت. به منظور مقایسه میانگین نمرات سلامت روانی دانشجویان، آزمون t-test دو گروهی مستقل به کار گرفته شد. داده های پژوهش با استفاده از نرم افزار SPSS مورد تجزیه و تحلیل قرار گرفت. نتیجه گیری: به طور کلی می توان گفت که دانشجویان دانشگاه لرستان از سلامت روانی بالایی برخوردار نیستند. علاوه بر آن دانشجویان دختر نسبت به پسران آسیب پذیر تر هستند و فعالیتهای گروهی با برخورداری از سلامت روانی ارتباط دارد. یافته ها: بررسی نمرات سلامت روانی کسب شده توسط دانشجویان با میانگین 19/164(کمی بالاتر از میانگین پرسشنامه با میانگین 150) نشان می دهد که برخی از آنان سلامت روانی پایینی دارند. یافته های پژوهش همچنین روشن ساخت که دانشجویان پسر نسبت به دختران، 99 درصد (01/0p<) از سلامت روانی بالاتری برخوردار هستند، سلامت روانی دانشجویان، ارتباطی با بومی و یا غیر بومی بودن آنان نداشت و نیز دانشجویانی که در فعالیتهای گروهی درون دانشگاه شرکت می کردند نسبت به دانشجویانی که در این گونه فعالیتها شرکت نمی کردند از سلامت روانی بالاتری برخوردار بودند (01/0p<).

Title: نتایج فانکشنال درمان جراحی شکستگی های رادیوس و النا

http://yafte.lums.ac.ir/article-1-1098-fa.html

مقدمه: شکستگی های جابجا شده استخوان های ساعد (رادیوس و النا) در بزرگسالان معمولاً توسط جا اندازی باز شکستگی و فیکساسیون داخلی درمان می شوند. هدف این مطالعه بررسی نتایج فانکشنال این شکستگی ها بود. نتیجه گیری: ریداکشن اناتومیک و فیکساسیون داخلی روش استاندارد درمان شکستگی های با جابجایی هر دو استخوان رادیوس و النای ساعد در بالغین است. با در نظر گرفتن یافته ها به نظر می رسد توجه بیشتر به انجام حرکات ساعد به خصوص پرونیشن و تقویت عضلات ساعد بعد از عمل جراحی در بهبود فانکشنال بیمار به خصوص پرونیشن و قدرت Grip تاثیر بسزایی داشته باشد. یافته ها: پرونیشن ساعد در مقایسه با سوپینیشن و در مقایسه با اندام سالم طرف مقابل کاهش یافته بود. دامنه حرکات فلکشن و اکستنشن مچ و آرنج به سطح نرمال قبل از عمل برگشته بود. قدرت Grip در مقایسه با طرف مقابل کاهش پیدا کرده بود. در پرسشنامه (DASH) نتایج رضایتبخش بودند. مواد و روش ها: در این مطالعه طی مدت پنج سال تعداد 52 بیمار که توسط جا اندازی باز رادیوس و النا و فیکساسیون داخلی توسط پلاکهای Dynamic compression plate (DCP) شماره 5/3 درمان شده اند مورد بررسی قرار گرفته اند. بعد از جراحی اندام به مدت یک هفته بی حرکت شده و سپس حرکات اکتیو آرنج و مچ دست و سپس تقویت عضلات ساعد بعد از دو ماه شروع شده است. مدت پیگیری 18 ماه (23-11) بوده و بیماران از لحاظ دامنه حرکات ساعد و مچ دست و قدرت گریپ گرفتن دست بررسی شدند. نتایج فانکشنال توسط پرسشنامه DASH بررسی شدند.

Title: بررسی بهره وری از دیدگاه پرستاران بالین و عوامل و موانع ارزشیابی در آن: تحقیقی کیفی

http://yafte.lums.ac.ir/article-1-1099-fa.html

مقدمه: پرستاران نقش اساسی در تداوم مراقبت و ارتقا سلامت دارند. بدین لحاظ بهره وری آنان اهمیت بسزایی در موفقیت سازمان دارد.گرچه عوامل زیادی بر بهره وری حرفه ها تاثیر دارد اما بررسی دیدگاه کارکنان حرفه اولین گام جهت پیشبرد بهره وری است. ضمن انکه روند و چگونگی ارزشیابی می تواند بعنوان عامل یا مانع بهره وری پرستاران عمل کند. نتیجه گیری: ارزشیابی موثر می تواند عامل موثری برای بهره وری پرستاران باشد بدین لحاظ اصلاحات اساسی در روند آن توصیه می گردد. یافته ها: تحلیل داده ها نشان داد پرستاران بهره وری را هم از جنبه کمی(کارایی) و هم از جنبه کیفی(اثربخشی) مورد توجه قرار می دهند؛ اما توجه غالب آنان بر اثربخش بودن مراقبتی است که به بیمار ارائه می دهند.همچنین از منظر آنان ارزشیابی موثر می تواند بهره وری پرستاران را ارتقا داده در حالی که موانع کنونی ارزشیابی محدودیتهایی را در ارتقا بهره وری تیم پرستاری ایجاد می نماید. مواد و روش ها: این تحقیق به روش کیفی انجام گردید. جهت جمع آوری اطلاعات از نمونه گیری هدفمند و مصاحبه های باز استفاده شد. داده ها از طریق تحلیل محتوا و به روش تحلیل مقایسه ای مداوم تجزیه تحلیل شدند.

Title: بررسی آناتومیکی شریان زیرچنبری و شاخه های آن

http://yafte.lums.ac.ir/article-1-1100-fa.html

مقدمه: شریان های زیرچنبری راست و چپ توسط انشعاباتی در خونرسانی نخاع گردنی، حفره جمجمه ای خلفی، قاعده گردن، غده تیروئید، جدار قفسه سینه و شکم نقش دارند و از برخی شاخه های آن مانند شریان سینه ای داخلی بعنوان مجرا در Bypass شریان های کرونر قلب در جراحی استفاده می شود. شاخه های این شریان در آنژیوگرافی، تشریح و جراحی های قاعده گردن و بخصوص غده تیروئید اهمیت دارد. با توجه به اینکه الگوی ثابتی برای واریاسیون ها وجود ندارد این بررسی انجام شده است. مواد و روش ها: در این مطالعه توصیفی- مقطعی، قاعده گردن 40 جسد فیکس شده، مذکر و با سن تقریبی 40 تا 80 سال با ابزاری مانند پنس، قیچی، تیغ بیستوری دو طرفه تشریح شدند و شاخه های شریان زیرچنبری تشریح و مشاهدات یادداشت شد. نتیجه گیری: حاصل این بررسی کشف جدیدی نیست؛ ولی با علم به این موضوع که واریاسیون ها الگوی ثابتی ندارند، توجه به شاخه های شریان زیرچنبری و واریاسیون های آن در بررسی تشخیصی، مطالعات آنژیوگرافی، جراحی های ناحیه گردن و بخصوص غده تیروئید اهمیت ویژه ای دارد. یافته ها: در 100% نمونه ها شریان زیرچنبری چپ از قوس آئورت و در 95% نمونه ها شریان زیرچنبری راست از شریان براکیوسفالیک منشعب شده بود. در یک نمونه(5/2%) از ابتدای قوس آئورت و در یک مورد(5/2%) از انتهای قوس آئورت جدا شده و به صورت رتروازوفاژیال قرار گرفته بود. در این بررسی شریان مهره ای چپ در دو نمونه و شریان مهره ای راست در یک نمونه واریاسیون داشت و از قوس آئورت جدا شده بودند. در بررسی ما شریان تیروسرویکال و شریان سینه ای داخلی واریاسیون نداشت.

Title: تعیین سطح سرمی منیزیم در کودکان مبتلا به تشنج ناشی از تب

http://yafte.lums.ac.ir/article-1-1101-fa.html

مقدمه: تشنج ناشی از تب شایعترین فرم تشنج در طول دوران کودکی است. این بیماری بیشتر در سنین 9 ماهگی تا 5 سالگی دیده می شود. تشنج ناشی از تب علت و پاتوژنزمشخصی ندارد، لذا بررسی علل احتمالی آن می تواند ارزشمند باشد..هدف از انجام این مطالعه بررسی و تعیین سطح سرمی منیزیم در اطفال 9 ماه تا 5 ساله مبتلا به تشنج ناشی از تب در مقایسه با اطفالی که صرفا مبتلا به تب بوده و دچار تشنج نشده اند. مواد و روش ها: در این مطالعه که به صورت علمی ـ مقایسه ای انجام شد،یک گروه 102 نفری از اطفال 9 ماه تا 5 سال مبتلا به تشنج ناشی از تب به عنوان گروه بیمار (مورد) و یک گروه 102 نفری از اطفال در همان گروه سنی که صرفا به علت تب مراجعه نموده واز لحاظ سن،رژیم غذایی، وزن و کلاخصوصیات فردی با گروه بیمارجور (Match) بودند به عنوان گروه کنترل در نظر گرفته شدند. متغیرهای مورد مطالعه شامل جنسیت،محل سکونت (شهری و روستا) ونوع آب آشامیدنی غالب بود.اطفالی که حائز شرایط انتخاب نمونه بودند از طریق روش نمونه گیری آسان وارد مطالعه شد. ابتدا پرسشنامه مربوط به طرح جهت آنها تکمیل و سپس نمونه سرمی آنها تهیه شده و سطح سرمی منیزیم آن از طریق روش سنجش بیوشیمیایی اندازه گیری شد. همین فرایند جهت گروه کنترل نیز انجام شد و درنهایت اطلاعات بدست آمده با استفاده از نرم افزار spss و تستهای آماری مورد تجزیه و تحلیل قرار گرفت.نتیجه گیری: پایین بودن سطح سرمی منیزیم در گروه بیمار نسبت به گروه کنترل می تواند بیانگر وجود رابطه بین سطح سرمی منیزیم و ابتلا به تشنج ناشی از تب در اطفال 9 ماه تا 5 سال باشد. پیشنهاد می شود مطالعات مشابهی با حجم نمونه بالاتر در مناطق مختلفی از ایران و جهان انجام گردد یافته ها: در کل بین میانگین سطح سرمی منیزیم در دو گروه اختلاف معنی داری بدست آمد. به طوریکه میانگین سطح سرمی منیزیم در گروه مورد به مراتب از میانگین سطح سرمی منیزیم در گروه کنترل پایین تر بود (0001/0p<). بین سطح سرمی منیزیم با جنسیت، در دو گروه ارتباط معنی داری بدست نیامد.همچنین بین سطح سرمی منیزیم با محل سکونت و نوع آب آشامیدنی غالب در هیچیک از دو گروه بیمار و شاهد ارتباط معنی داری بدست نیامد. بین سطح سرمی منیزیم وسن اطفال نیز در هیچیک از دو گروه ارتباط معنی داری بدست نیامد. یک نکته جالب در این پژوهش این بود که میانگین سطح سرمی منیزیم در هر دوگروه نسبت به دامنه نرمال به طور معنی داری بالاتر بود.

Title: ارتباط بین هلیکوباکترپایلوری و سطح سرمی ویتامین ب12 در خانمهای سنین باروری

http://yafte.lums.ac.ir/article-1-1102-fa.html

مقدمه: عفونت هلیکوباکترپایلوری یکی از علل مهم مشکلات گوارشی بخصوص زخم معده است که اخیرا" نشان داده شده یک ارتباط معنی دار بین کاهش سطح سرمی ویتامین ب12 و سطح سرمی هلیکوباکترپایلوری وجود دارد. هدف ازاین مطالعه بررسی ارتباط بین سطح سرمی ویتامین ب12 و سطح سرمی هلیکوباکترپایلوری بود. مواد و روش ها: مطالعه حاضر از نوع توصیفی ، مقطعی بود که از سال 1382 تا سال 1383 بر روی 110 نفر از خانمهای سالم بدون سابقه بیماری در محدوده سنی 49-15 سال مراجعه کننده به درمانگاههای شهر خرم آباد انجام گرفت. که هر نمونه از نظر سطح سرمی ویتامین B12,CBC و آنتی بادی هلیکوباکترپایلوری مورد بررسی قرار گرفتند. اطلاعات فردی -سابقه بیماری (بخصوص بیماری گوارشی) آنان در پرسشنامه ثبت شدند و اطلاعات بدست آمده توسط نرم افزار SPSS مورد بحث و تحلیل قرار گرفتند. نتیجه گیری: شیوع بالای عفونت هلیکوباکترپایلوری بین نمونه های با سطح سرمی پائین ویتامین ب12 نشان می دهد که یک ارتباط معنی دار بین هلیکوباکترپایلوری و سطح ویتامین ب12 وجود دارد. یافته ها: نتایج بدست آمده نشان داد از بین 110 نمونه مورد مطالعه 76 نمونه (72.2%) سطح سرمی هلیکوباکترپایلوری آنان مثبت بود و سطح سرمی هلیکوباکترپایلوری بطور معنی داری در افرادیکه دارای میزان ویتامین ب12 کمتر ازحدود طبیعی بوده ، بالاتر بود، به طوریکه بین 54 نمونه با سطح سرمی ویتامین ب12 کمتر از pg/mg145 و سطح متوسط ml/pg180-145 ، سطح آنتی بادی هلیکوباکترپایلوری مثبت بود (0.0001 p<).

Title: فراوانی موراکسلا کاتارالیس catarrhalis) (Moraxella و ریسک فاکتورهای ابتلاء به آن در بیماران مبتلا به عفونتهای تنفسی مراجعه کننده به بیمارستانهای ولیعصر و امیرکبیر اراک

http://yafte.lums.ac.ir/article-1-1103-fa.html

مقدمه: موراکسلاکاتارالیس دیبلوکوک گرم منفی است که می‌تواند عامل عفونتهای تنفسی تحتانی مانند برونشیت، پنومونی و عفونتهای تنفسی فوقانی بخصوص عفونت حاد گوش میانی و سینوزیت باشد. با مراجعه به آزمایشگاههای سطح شهرستان اراک مواردی از جداسازی و انجام آنتی بیوگرام این باکتری مشاهده نگردید. بنابراین دلایل، این تحقیق پایه ریزی گردید تا ضمن آشنایی همکاران بالینی و آزمایشگاهی با این باکتری, بتوان با عمل آنتی بیوگرام گامی مؤثر در جهت درمان سریع و مناسب بیماران مبتلا به این نوع عفونت‌ها برداشت. مواد و روش ها: این مطالعه از نوع توصیفی تحلیلی از شهریور لغایت اسفند 1382 و بر روی 200 بیمار مراجعه کننده به بیمارستانهای ولیعصر و امیرکبیر اراک صورت گرفت. نمونه ها بعد از بررسی مستقیم و کشت توسط روشهای میکروبیولوژیک مورد تایید قرار گرفته سپس حساسیت و مقاومت آنها نسبت به آنتی بیوتیکها ارزیابی گردید و نوع مقاومت به پنی سیلین نیز مشخص شد. نتیجه گیری: با استناد به یافته‌های این تحقیق موراکسلاکاتارالیس باکتری پاتوژنی است که در عفونتهای تنفسی تحتانی (بخصوص افراد مبتلا به بیماری انسدادی مزمن ریه و مسن) و در عفونتهای تنفسی فوقانی (عفونت حاد گوش میانی و سینوزیت) می بایستی همواره مد نظر قرار گیرد و جهت مقابله صحیح با عفونتهای این باکتری و جلوگیری از افزایش مقاومت آنتی بیوتیکی باید در هر ناحیه جغرافیایی مطالعه کامل صورت پذیرد و استراتژی صحیح بر خورد با آن تدوین گردد. یافته ها: از 200 بیمار مورد بررسی در مجموع 17 مورد(5/8%) موراکسلاکاتارالیس ایزوله گردید، بطوریکه از 123 بیمار مبتلا به عفونت تنفسی تحتانی 11 مورد (9/8%) و از 42 بیمار مبتلا به عفونت حاد گوش میانی 4 مورد (5/9%) و در عفونت سینوزیت از 35 نمونه 2 مورد (7/5%) باکتری ایزوله گردید.

Title: معرفی دو مورد نادر موکوسل آپاندیس در بیمارستان گلستان اهواز

http://yafte.lums.ac.ir/article-1-1104-fa.html

مژگان ساکی3 مقدمه: موکوسل آپاندیس یک اصطلاح غیر اختصاصی است که برای توضیح آنومالی آپاندیس که با موکوس اتساع پیدا کرده اطلاق می شود که ممکن است در نتیجه علل نئوپلاستیک یا غیر نئوپلاستیک ایجاد شود. در این مطالعه دو مورد نادرموکوسل آپاندیس معرفی می شود نتیجه گیری: بدخیمی آپاندیس بی نهایت نادر است، 2 مورد جدید فوق با توده شکمی و درد مراجعه نمودند و موکوسل خوش خیم با آپاندکتومی ساده درمان می شود. معرفی بیماران: بیمار اول خانم 70 ساله ای بود که با شکایت شکم درد از یک سال قبل که از سه ماه پیش تشدید شده بود.عمــــده درد بیمار در قسمت تحتانی شکم بوده،بیمــار کاهش وزن _ کاهش اشتها نداشته، از یبوست شاکی بوده سابقه کله سیستکتومی 14 سال پیش را می داد. سابقه دیابت و بیماری ایسکمیک قلب را از سه سال پیش داشته است. در معاینه یک توده توپری در 4/1 تحتانی راست شکم و پهلوی راست داشت.در بررسی تکمیلی، سونوگرافی یک توده کیستیک با اجزا توپر در پایین پل تحتانی کلیه راست با تشخیص کیست مزانتریک، کیست هیداتید، توده بد خیم گزارش کرده بود در بررسی CT اسکن Duplication cyst کیست مزانتریک و کیست کلیوی گزارش شده بود. بیمار دوم آقای 38 ساله ای بود با شکایت شکم درد شکم از 5 روز قبل مراجعه کرده بود که شروع درد در اپی گاستر بوده و سپس به 4/1 تحتانی راست شکم کشیده شده بود.بیمار از انتشار درد به پهلوی راست شاکی بود.بی اشتهایی را ذکر می کرد.در بررسیهای تکمیلی لکوسیتوز 11000 با 64% داشت. در بررسی سونوگرافی، یک توده کیستیک با انترنال اکو در ناحیه تحتانی راست شکم و آبسه آپاندیکولر مشاهده شده بود. بیماران فوق تحت عمل جراحی قرار گرفتند، بیمار اول لاپاراتومی شد که موکوسل آپاندیس بود و بقیه شکم نیز بررسی شد که نرمال بود و بیمار دوم نیز با انسزیون مک بورنی باز شد که باز هم موکوسل آپاندیس بود. آبسه وجود نداشت، آپاندکتومی در هر دو مورد انجام شد و در بررسی پاتولوژی هر دو مورد موکوسل آپاندیس بود.
